# Difference in Patient Outcomes Coming From Public and Private Hospitals in an Intensive Care Unit in Brazil

**DOI:** 10.4021/jocmr1157w

**Published:** 2012-11-11

**Authors:** Fabio F Amorim, Adriell R Santana, Rodrigo S Biondi, Alethea PP Amorim, Edmilson B Moura, Karlo J Quadros, Humberto S Oliveira, Rubens AB Ribeiro

**Affiliations:** a Faculty of Medicine, Escola Superior de Ciencias da Saude, Brasilia, DF, Brazil; b Adult Intensive Care Unit, Hospital Anchieta, Brasilia, DF, Brazil; c Centro de Ensino e Tecnologia em Ciencias da Saude, Brasilia, DF, Brazil; d Adult Intensive Care Unit, Hospital de Base do Distrito Federal, Brasilia, DF, Brazil

**Keywords:** Intensive care units, Health inequalities, Outcomes, Mortality, National health system, Private health system

## Abstract

**Background:**

Compare demographic data, mortality and intensive care unit length of stay (ICU LOS) in patients coming from public hospitals of the Brazilian Unified Health System and patients coming from private hospitals of the Brazilian Supplementary Health System in a single private general ICU.

**Methods:**

A retrospective cohort study was performed on patients in the ICU of Hospital Anchieta in Brasilia, DF, Brazil, over a period of 2 years. The patients were divided into 2 groups: patients from public hospitals of the Unified Health System group (PUBH, N = 75) and patients from private hospitals of the Brazilian Supplementary Health System group (PRIH, N = 1,614).

**Results:**

In total, 1,689 patients were admitted. For the entire cohort, the median age was 62 ± 17 years, and the mean APACHE II score was 13 ± 7. The PUBH had a higher APACHE II score (18 ± 9 versus 12 ± 7, P = 0.00), were younger (53 ± 2 versus 63 ± 16 years, P = 0.00), and had higher incidence of circulatory shock (19.2 versus 11.4%, P = 0.01), and kidney injury or renal failure (38.4 versus 25.5%, P = 0.01) at the time of ICU admission, compared to the PRIH. The ICU LOS was longer for the PUBH compared to the PRIH (18 ± 18 versus 6 ± 14 days, P = 0.00). The overall mortality rate was higher for the PUBH compared to the PRIH (33.3 versus 9.7%, P = 0.00).

**Conclusions:**

In a single ICU, where patients had access to the same human and technological resources, patients from the PUBH had a higher APACHE II score, ICU LOS, and mortality rate than those from the PRIH.

## Introduction

The Brazilian Health System consists of a complex network of a public sector, called the Unified Health System (SUS), which is financed by the state at the federal, state, and municipal levels, and a private sector, called the Supplementary Health System, which is financed by employers and/or households and includes group medicine, medical cooperatives, and insurance companies [1, 2]. The Constitution of the Federative Republic of Brazil in 1988 stated that the Brazilian Health System should be based on the principles of equality, universality, and comprehensive care for all Brazilian citizens [3]. However, as was observed in other countries that have adopted a similar model, the existence of an additional private sector brings a great challenge, which is the inequality in access to health care [1, 2, 4].

Patients with access to private services have higher-quality health care, which can be observed in the intensive care units (ICUs), where studies have shown better outcomes for private hospitals compared to public hospitals [5, 6]. The private hospitals compared to the public hospitals usually have greater access to technological and human resources. This factor has been implicated as the cause of this difference in performance [5]. However, there are no studies that compare the outcomes of patients from public and private hospitals admitted to a single ICU, where they had access to the same human and technological resources.

The main purpose of this study was to compare the mortality of patients coming from public and private hospitals admitted to a single ICU. Simultaneously, we assessed the intensive care unit length of stay (ICU LOS), demographic data, and the presence of circulatory shock, and kidney injury or renal failure at the time of ICU admission.

## Methods

### Study design and setting

This was a retrospective cohort study conducted from January 2008 to December 2009, involving all adult patients admitted consecutively to the ICU of Hospital Anchieta, a tertiary care private hospital with 140 beds and 24 ICU beds ICU, located in Brasilia, DF, Brazil.

The Ethics Committee of Hospital Anchieta approved the study and waived the need for informed consent. The conduction of the study did not interfere with patient management decisions.

### Subjects

All of the patients were admitted to the ICU during the study period. Patients younger than 18 years old, transferred from another ICU, readmitted during the same hospitalization, or transferred to another ICU were excluded from the study.

### Measurements and outcome evaluation

Patients were divided into two groups: patients coming from public hospitals of the Unified Health System group (PUBH) and patients coming from private hospitals of the Supplementary Health System group (PRIH).

All of the patients had an APACHE II score computed within 24 hours of admission. Each participant was followed until the ICU discharge or death. We also recorded demographic data, mortality, ICU LOS, and the presence of circulatory shock, and kidney injury or renal failure according to the RIFLE classification [7] at the time of ICU admission.

### Statistical analysis

The Kolmogorov-Smirnov test was used to assess the normality of the data. Continuous variables with a normal distribution were compared using Student’s t-test. Continuous variables without a normal distribution were compared using the Wilcoxon-Mann-Whitney U test. Categorical parameters were compared using the Chi-square test (χ^2^). The level of significance was set at 5% (P < 0.05). All of the analyses were performed using the Statistical Package for Social Sciences 17.0 for Windows (SPSS 17.0 for Windows, SPSS Inc., Chicago, Illinois, USA).

## Results

Among the 1,689 patients included in the study, 183 patients came from public hospitals of the Brazilian Unified Health System (11%) and 1,467 patients came from private hospitals of the Brazilian Supplementary Health System (89%). For the entire cohort, the mean APACHE II score was 13 ± 7, the median age was 62 ± 17 years, and 935 patients were male (55.4%). The PUBH had a higher APACHE II score (18 ± 9 versus 12 ± 7, P = 0.00), were younger (53 ± 22 versus 63 ± 16 years, P = 0.00), and had a higher incidence of circulatory shock (19.2 versus 11.4%, P = 0.04), and kidney injury or renal failure (38.4 versus 25.5%, P = 0.01) at the time of ICU admission compared to the PRIH. There were more admissions for a medical diagnosis from the PUBH than the PRIH (97.3 versus 51.3%, P = 0.00) (Table 1). The ICU LOS was longer for the PUBH compared to the PRIH (18 ± 18 versus 6 ± 14 days, P = 0.00). The mortality rate was higher for the PUBH compared to the PRIH (33.3 versus 9.7%, P = 0.00) (Table 2). Analyzing only the medical patients, the PUBH still had a higher APACHE II score (18 ± 9 versus 15 ± 8, P = 0.00) and were younger (52 ± 22 versus 64 ± 18 years, P = 0.01) compared to the PRIH. At the time of ICU admission, the PUBH had a higher incidence of circulatory shock (19.7 versus 11.3%, P = 0.03) compared to the PRIH, but there were no difference between the groups regarding kidney injury or renal failure (43.7 versus 43.0%, P = 0.51) (Table 3). The ICU LOS was longer for the PUBH compared to the PRIH (18 ± 18 versus 9 ± 18 days, P = 0.00). The mortality rate was higher for the PUBH compared to the PRIH (33.8 versus 17.1%, P = 0.00) (Table 4).

**Table 1:** Baseline Demographics of Patients in the Unified Health System Group (PUBH) and the Supplementary Health System Group (PRIH)

		PUBH	PRIH	P-value
		(N = 75)	(N = 1,614)	
Age (years)	Mean (SD)	53 (22)	63 (16)	0.00^*^
APACHE II	Mean (SD)	18 (9)	12 (7)	0.00^*^
Male	%	45.3	55.8	0.05
Medical diagnosis	%	97.3	51.3	0.00^*^
Kidney injury or renal failure	%	38.4	25.5	0.01^*^
Circulatory shock	%	19.2	11.4	0.04^*^

The differences were compared using the Student’s t-test, Wilcoxon-Mann-Whitney U test, and Chi-square test (χ^2^) where appropriate. ^*^Statistical significance P < 0.05. APACHE II = Acute Physiology and Chronic Health Evaluation II, SD = Standard Deviation.

**Table 2:** Outcomes of Patients in the Unified Health System Group (PUBH) and the Supplementary Health System Group (PRIH)

		PUBH	PRIH	P-value
		(N = 75)	(N = 1,614)	
ICU LOS (days)	Mean (SD)	18 (18)	6 (14)	0.00^*^
Mortality	%	33.3	9.7	0.00^*^

The differences were compared using the Student’s t-test and chi-square test (χ^2^) where appropriate. ^*^Statistical significance P < 0.05. ICU LOS = intensive care unit length of stay, SD = Standard Deviation.

**Table 3:** Baseline Demographics of Patients With a Medical Diagnosis in the Unified Health System Group (PUBH) and the Supplementary Health System Group (PRIH).

		PUBH	PRIH	P-value
		(N = 73)	(N = 836)	
Age (years)	Mean (SD)	52 (22)	64 (18)	0.01^*^
APACHE II	Mean (SD)	18 (9)	15 (8)	0.00^*^
Male	%	45.2	53.5	0.18
Kidney injury or renal failure	%	43.7	43	0.51
Circulatory shock	%	19.7	11.3	0.03^*^

The differences were compared using Student’s t-test, Wilcoxon-Mann-Whitney U test, and chi-square test (χ^2^) where appropriate. ^*^Statistical significance P < 0.05. APACHE II = Acute Physiology and Chronic Health Evaluation II, SD = Standard Deviation.

**Table 4:** Outcomes of Patients With a Medical Diagnosis in the Unified Health System Group (PUBH) and the Supplementary Health System Group (PRIH).

		PUBH	PRIH	P-value
		(N = 73)	(N = 836)	
ICU LOS (days)	Mean (SD)	18 (18)	9 (18)	0.00^*^
Mortality	%	33.8	17.1	0.00^*^

The differences were compared using the Student’s t-test and Chi-square test (χ^2^) where appropriate. ^*^Statistical significance P < 0.05. ICU LOS = intensive care unit length of stay, SD = Standard Deviation.

## Discussion

In this study of admissions in a single ICU, patients coming from the SUS were younger but presented with more severe disease compared to patients coming from the Supplementary Health System at the time of admission. There was also a higher rate of circulatory shock, and kidney injury or renal failure among patients coming from the SUS, who had a longer hospital stay and higher mortality rate compared to patients coming from the Supplementary Health System. Hospitalization for a medical diagnosis was more common in patients from the SUS, which accounted for almost all of the cases in this group (97.3%). Thus, we opted to conduct an analysis only with patients with a clinical diagnosis. Even in this analysis, patients from the SUS had a higher mortality rate and ICU LOS compared to patients coming from the Supplementary Health System.

Silva and colleagues published data from a cohort study [5] conducted in patients hospitalized for sepsis in five mixed ICUs of private and public hospitals in Brazil. That study observed a higher mortality for patients from the public hospitals compared to those from private hospitals, even if no significant differences were observed in the APACHE II score of these patients, suggesting that these differences could be related to factors including the differences in technological and human resources between the private and public hospitals.

Another study performed by Ferreira and colleagues [6] comparing mortality and morbidity in patients with acute myocardial infarction hospitalized in public and private hospitals in Feira de Santana, BA, Brazil observed that patients in the private hospitals showed a markedly low mortality rate, comparable to the rates in countries with a high per capita income. However, the mortality rate in public hospitals was high, even above the mortality rate in countries with a worse per capita income than Brazil.

Nevertheless, this fact is similar to that observed in other Latin America countries, such as Colombia, where a survey study conducted by Perez and colleagues [8] in 20 ICUs showed a marked difference between the public and private sectors, where all four of the ICUs with the lowest mortality ratio belonged to private hospitals, while four of five ICUs with the highest mortality belonged to public hospitals.

Unlike these previous studies, this study was conducted in a single ICU with patients from the public and private sectors, who had access to the same technological and human resources. This suggests that other factors before ICU admission are also involved in this phenomenon. It is known that various conditions can influence patient outcomes in the ICU setting, including the delay to ICU admission and pre-existing conditions, such as nutritional status [9-14].

An important factor is the time elapsed between the admission request and ICU admission [14-18]. It has been shown that the delay in establishing specialized care affects patient outcomes, especially when adequate treatment is not instituted quickly as early goal-directed therapies [17-18]. Furthermore, there is a window of opportunity to implement these treatments. Additionally, patient transfers to the ICU do not lead to improved patient outcomes [19]. In a study designed to assess the impact of delayed patient transfer from the emergency department to the ICU, Chalfin and colleagues [15] showed that patients who take more than six hours to be transferred to the ICU had an increased hospital length of stay and higher ICU and hospital mortalities. A similar result was observed in a study conducted in Brazil [16]. Another Brazilian study conducted on surgical patients by Chiavone and Rasslan [14] has observed that the delay between the end of a surgery and ICU admission was associated with a worse APACHE II score and mortality rate. All of these factors must be related to the findings of our study.

A positive aspect of our study was the ability to compare the clinical outcomes of the patient groups in which the monitoring and the treatment have been standardized in a single ICU because, otherwise, the initial treatments would most likely have been so different. Thus, we conclude that the impact on patient morbidity and mortality can be attributed not only to the quality of care in an ICU but also to other factors, such as the health system in which the ICU is located. It is well known that, in public hospitals in Brazil, the conditions for the appropriate treatment of critically ill patients are insufficient due to the high influx of patients, lack of resources, and lack of trained staff [20]. Measures to ensure an appropriate early treatment in an emergency environment for critically ill patients could result in significant decreases in mortality and a subsequent reduction in costs. Recent studies have shown that training and supporting appropriate emergency professionals in the care of critically ill patients, especially patients with severe sepsis or septic shock, to be cost effective [21-22].

### Conclusion

In a single intensive care unit, where the patients had access to the same human and technological resources, the PUBH had a higher APACHE II score and mortality compared to the PRIH. Thus, we conclude that morbidity and mortality of these patients can be attributed not only to the quality of care in an ICU but also to other factors, such as the health system in which the ICU is located. Future studies are needed to investigate the determinants of these findings, such as the social and economic aspects and the delay to ICU admission.

## Declaration

This work was performed in the Adult Intensive Care Unit at Hospital Anchieta in Brasilia, DF, Brazil.
